# Agricultural Cold Chain Logistics Mode Based on Multi-Mode Blockchain Data Model

**DOI:** 10.1155/2022/8060765

**Published:** 2022-06-27

**Authors:** Yang Si

**Affiliations:** Economics and Management School, Qujing Normal University, Qujing, Yunnan 655011, China

## Abstract

Aiming at the problem of massive data storage and transaction authority in the blockchain system in the data sharing transaction scenario, this paper proposes a blockchain system for the controllable sharing transaction of massive data. By designing the blockchain system model of the consortium chain and private chain, reconstructing the data structure of the block, and introducing the InterPlanetary File System (IPFS) technology, we adopt the off-chain storage method to solve the problem of high cost caused by too much data carried by the block. The isolation control of data resource transactions is realized by introducing identity authentication and channel mechanism, which meets the needs of different subjects for confidentiality of transaction data information. From the analysis results of cold chain logistics efficiency, in the five years from 2017 to 2021, the comprehensive cold chain logistics efficiency, pure technology cold chain logistics efficiency, and scale cold chain logistics efficiency of agricultural product cold chain logistics in a certain region have all reached above 0.9. The return to scale remains unchanged, and the cold chain logistics resources have been effectively allocated and reasonably utilized. The value of comprehensive cold chain logistics efficiency, pure technology cold chain logistics efficiency, and scale cold chain logistics efficiency in a certain month is less than 1, which means that the cold chain logistics efficiency is weak or ineffective. This shows that in these years, the infrastructure investment, cold chain technical support, and management level related to the cold chain logistics of agricultural products in a certain region need to be strengthened, and it is necessary to adjust the input-output structure and allocate resources reasonably. From 2018 to 2021, the total factor productivity of agricultural product cold chain logistics in a certain region is greater than 0.85, indicating that the level of total factor productivity is relatively high during this time period, and the increase in the efficiency of technical cold chain logistics is the main reason for the increase in total factor productivity.

## 1. Introduction

In the context of rapid economic development, the per capita income of the people is rising, the living standards of residents are steadily improving, the people are paying more and more attention to the safety of food, the consumption concept is also changing constantly, and cold chain logistics has entered a new period of development [1]. In order to provide residents with quality and safe products and reduce the damage rate of food during transportation, it is urgent to improve cold chain logistics. The latest data show that the annual loss rate of perishable food in China is as high as 20%–30% [2]. With the acceleration of urbanization, the consumption of perishable products in urban areas is as high as 76%. Today, about 400 million tons of agricultural products circulates in the market every year, and the proportion of cold chain transportation continues to rise. The cold chain circulation rates of fruits and vegetables, fresh meat, and aquatic products are 5%, 15%, and 23%, respectively [3]. At present, the modern logistics system for agricultural and sideline products in China has not yet been established, and the development of cold chain logistics is also slow. The production of agricultural products is still dominated by the decentralized production of each farmer. The supply and marketing cooperative basically does not live up to its name, and it has very little effect on the transportation and sales of agricultural products, especially fresh agricultural products. The slow development of circulation channels restricts the mass production of fresh and live agricultural products, and also affects the storage and transportation [4].

Blockchain is a decentralized distributed ledger technology and a distributed database. With the birth of Bitcoin, blockchain uses P2P dynamic real-time transmission of network information, encryption technology, hash algorithm, digital signature technology, and consensus authentication mechanism to solve the nonrepudiation of blockchain-based business activities [5]. The problem has promoted the development of smart contract application research. This realizes the sharing of information and the dynamic instantaneousness of transmission, as well as the decentralized dynamic supervision of business activities, and promotes the realization of group decision-making in the process of business content management. In the blockchain system, other business entities can conduct dynamic real-time supervision and interaction on the sales behavior and distribution activities of sellers, which improves the cold chain logistics efficiency of sales management and logistics distribution; because the blockchain has a strong business reorganization capacity, the flexibility of supply chain business entities to enter and exit the supply chain has improved, so the blockchain promotes the competition of similar business entities in the supply chain and optimizes the structure of supply chain members; in addition, the supply chain provides the security guarantee that the information is authentic and indestructible [6].

The application of blockchain technology to the cold chain logistics industry is conducive to enhancing the trust between all parties involved in the logistics process. Blockchain technology has the characteristics of multi-party maintenance and distributed storage. In the process of cargo transportation, all parties have equal status and jointly own all the data on the chain. All information on the chain is open and transparent, forming a safe and reliable data sharing platform. It is conducive to the confirmation and traceability of goods assets. Blockchain technology has the characteristics of tamper-proof and traceability. All asset data during the transportation of goods are completely recorded in the blockchain network, and the blockchain ledger data have timestamps, writers, and other information. The ledger data of each participant are the same, and the ledger data cannot be changed or deleted, thus realizing the confirmation and traceability of the goods assets. The application of blockchain technology to the cold chain logistics industry is conducive to accelerating the research on the implementation of blockchain technology. The implementation of blockchain technology is still in the initial stage of exploration [7]. This research can accumulate useful experience for the implementation of blockchain technology, use blockchain technology to improve the efficiency of cold chain logistics for the real economy, and promote the standardized development of traditional industries.

Compared with the existing public chain models of Bitcoin and Ethereum, this blockchain ensures that the transaction information of transaction participants will not be queried by third parties by designing an access control mechanism, effectively enhancing the privacy and security of the blockchain platform. By combining IPFS technology and adopting off-chain storage, the storage consumption of the blockchain is effectively reduced. Through empirical analysis, it can be found that from 2017 to 2021, the cold chain logistics efficiency of agricultural products in a certain region in 2018 was close to 1, reaching the DEA effective state, and the cold chain logistics efficiency in other years was DEA effective. Compared with other provinces in the country, the comprehensive cold chain logistics efficiency level of agricultural products in a certain region is higher, and the average value of cold chain logistics efficiency is larger, indicating that the investment has achieved better utilization. After decomposing the comprehensive cold chain logistics efficiency, it is found that the higher efficiency of pure technology cold chain logistics is the main reason for the better level of logistics cold chain logistics efficiency. It is comprehensively explained that there are reasonable resource allocation, high technical level, and high management level in the cold chain logistics of agricultural products in a certain region, which makes the cold chain logistics of agricultural products in a certain region more efficient.

## 2. Related Work

In order to optimize the cold chain logistics system of agricultural products, the researchers started from the perspective of cold chain logistics companies, such as using IoT devices to collect information such as the temperature in the cold chain logistics compartment [8]. Since IoT can make devices intelligent and realize automatic transmission and measurement instead of manual operation, the use of IoT devices can not only save labor costs, but also improve the reliability of data. However, the product information in the cold chain is stored in the centralized database of the logistics company. Since the centralized database is private, it not only has the risk of single point of failure and is easy to be tampered with, but also causes certain difficulties for the supervision of the regulatory authorities.

In order to reduce the loss of agricultural products during transportation, some researchers, from the perspectives of regulators and consumers, solve the difficulties caused by the storage of logistics data in a private centralized database and use blockchain to store data in logistics [9]. Due to the open, transparent, and difficult to tamper characteristics of the blockchain, it not only makes the data more credible, but also facilitates the supervision of the regulatory authorities. However, the penalties imposed by the regulatory authorities on illegal logistics companies are delayed, which may cause some logistics companies to still try the law [10].

JD.com released a white paper based on blockchain application research, using the form of blockchain alliance chain to try to solve the problem of anti-counterfeiting traceability of JD.com and the company's products, and to establish a research laboratory based on blockchain application system platform. Based on the blockchain and combined with its technical accumulation in data mining and intelligent logistics, they build an integrated platform integrating intelligent logistics system, offline sales, and big data analysis, and improve the core competitiveness of enterprises in product traceability and anti-counterfeiting issues [11].

Relevant scholars proposed to build a supply chain information platform based on blockchain technology, combining government supervision departments with the financial industry and other industries, so that the business flow, goods flow, and capital flow in the supply chain can be gathered into one platform, so as to create a mutually trusted supply chain system [12, 13].

The researchers analyzed the difference between financial service platforms and traditional finance, built the business model of the “blockchain + logistics finance” platform, and realized the prediction and control of platform business risks and the supervision of the entire logistics system through blockchain technology [14]. Relevant scholars have proposed a blockchain technology solution for the cold chain logistics industry, which will combine the decentralization and trustworthiness of blockchain technology to securely store the circulation data in the cold chain physical industry into the blockchain [15]. It improves the reliability and data security of the cold chain logistics industry.

Related scholars have proposed a distributed solution that combines blockchain technology with traditional cloud servers [16]. Instead of registering the drone itself to the blockchain, it records the hash data collected from the drone. Anchoring to the blockchain network and generating a blockchain receipt for each data record stored in the cloud reduces the burden on drones by limiting battery and processing power, while gaining enhanced data security guarantees [17].

In order to solve the problems of centralized storage, easy tampering, and poor security in archival data management, relevant scholars have proposed a decentralized archive data protection and sharing method using blockchain-related technologies [18]. Technologies such as interplanetary file systems, digital signatures, and smart contracts are conducive to promoting the transformation of archive data storage methods [19]. Relevant scholars have realized the access control of data within enterprises and the secure sharing of data between enterprises based on blockchain technology, and used attribute-based encryption to control and share enterprise data, so as to achieve the purpose of fine-grained access control and safe sharing [3].

## 3. Methods

### 3.1. The Overall Architecture of the Data Model

**Figure 1:**
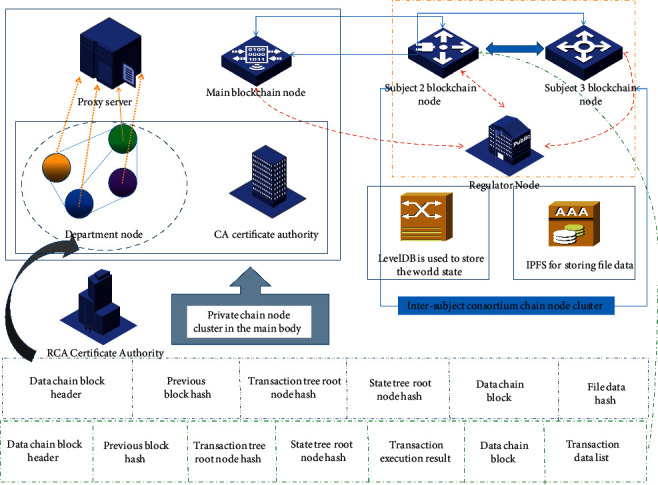
Overall architecture design diagram of the data model.

The overall architecture design of the blockchain data model is shown in Figure 1. The figure generally includes the certification body RCA of the alliance chain, the network deployment of each node between and within the main body of the alliance chain, data storage, and the block structure of the alliance chain and private chain.

As shown in Figure 1, there is a root certificate authority (RCA) in the alliance chain, and each subject has its own certificate authority. The certificate authority uses the form of certificate trust chain to ensure the legality of authorization at all levels. The alliance chain block responsible for transactions mainly stores transaction information, and the private chain block responsible for data storage is mainly responsible for storing data files. The storage of data files is in the form of off-chain storage, so the files are actually stored in the IPFS network built by the alliance organization, and LevelDB is used to store the world state of the ledger. The following subsections will explain in detail the five aspects of data model architecture design, business smart contract design, rights management and identity authentication design, access control mechanism design, and data storage structure design.

### 3.2. Double-Chain Construction Model

This model is mainly designed based on the consortium chain, which is suitable for the scenario of data sharing and transactions between multiple organizations. Constructing a private chain within the main body is responsible for storing data information.

**Figure 2:**
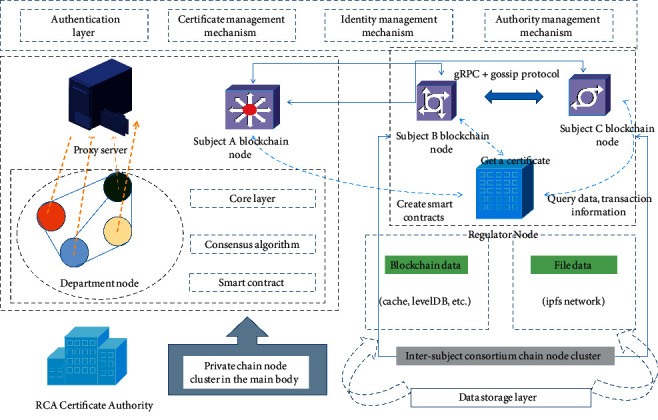
Double-chain construction model diagram.

Figure 2 shows a double-chain model, which is mainly composed of a consortium chain and a private chain. In the consortium chain, each main node is the center, forming a multi-center blockchain structure from a macro perspective. First, we build a private chain network within the main body. Within the main body, different departments of the main body can form a node cluster. Different departments can realize the sharing of data and information within the main body through the private chain. At the same time, due to the characteristics of the blockchain, it can avoid data tampering and data tampering caused by centralized data storage. Server fault-tolerant maintenance costs are too high, and data sharing and dissemination cold chain logistics efficiency is too low. Since the private chain is deployed in the local area network and is isolated from the Internet, the security of the private data within the subject can be guaranteed through physical isolation. The certificate authority (CA) is used to provide identity certificates and transaction certificates for the nodes participating in the transaction to verify the identity of the counterparty and the legitimacy of the transaction.

The consortium chain and the private chain network are isolated through a proxy server, and the docker image of Nginx is installed in the server as a proxy server. The proxy server itself is in the private chain network and is managed and maintained by each subject. Using the proxy and load balancing functions of the Nginx proxy server, the data transaction information is transmitted between the private chain node and the alliance chain node through the proxy server to realize the data transaction between the alliance chains.

The CA agency is mainly responsible for reviewing the identity of the subject and performing operations such as admission and clearance. The inter-subject node is responsible for the transaction, and the alliance chain is responsible for storing the transaction data. The transaction initiator has the right to conduct related transactions after being authorized and authenticated by the CA. When initiating a transaction, steps such as transaction verification and consensus sorting are also required. After the transaction is completed, the node transaction information will be processed. Accounting processing, this blockchain model supports the gossip network protocol, and each node communicates through *g* RPC.

### 3.3. Business Smart Contract Design

Because most subjects do not want the data information, they participate in the transaction to be seen by all other organizations, and privacy protection is a problem that we need to consider when designing a blockchain data sharing transaction platform. Based on this, this paper proposes a multi-layer blockchain architecture model. The previous section mainly analyzed the layered structure and architecture design of the data model. This section mainly analyzes the business processing of the two types of chains.

The private chain only stores data files and user information, and does not execute transactions. It is built within the main body, uses the main internal LAN for communication, and is isolated from the external Internet, ensuring the security of the main data. This platform uses the IPFS protocol to store real files. The block of the private chain stores the hash value of the file processed by IPFS technology. The IPFS client or browser can use the IPFS client or browser to obtain the file data according to the hash value. This section is temporarily understood as the private chain can carry data files. The business smart contract design of the private chain is as follows: first, users can upload data files through the private chain system. Since the data file information is stored on all nodes of the network through the platform, this model can ensure that the data information is difficult to be tampered with. Users can query, add, delete, and modify file data information through smart contracts. At the same time, the distributed data storage method can enhance the disaster tolerance of the system and reduce system maintenance costs. The data designed by the business smart contract are divided into user information, data information, and operation information. The specific content is shown in Table 1.

User information mainly indicates the relevant information of an account, the department name indicates the department the account belongs to in the main body, and the account public key, account private key, and account address are necessary elements for the existence of an account in the blockchain platform.

The data information mainly represents the uniqueness of the file in the file system. According to the characteristics of the IPFS protocol, different files have different file hash values, so the uniqueness of the file data can be judged according to the hash value, and the file account number indicates the uploader of the file.

The operation information mainly records the operation types of different users on the data file, which are divided into adding, querying, deleting, and modifying the file data. The private chain mainly realizes the interaction of information between users within the main body.

Table 2 shows the data structure of the alliance chain business smart contract, in which the transaction information is mainly the address of the initiating party, the address of the accepting party, the transaction time, and the transaction status; the operation information is the operation number, the account performing the operation, the operation generation time, and operation type. Since the alliance chain is only responsible for transactions, and the data are stored on the private chain, when the subject needs to initiate a transaction, the data information needs to be shared on the alliance chain.

### 3.4. Complete Transaction Process

#### 3.4.1. Transaction Endorsement

The client initiates a transaction and sends a request to any endorsing node related to this transaction. After the endorsement node receives the request, it simulates the execution of the smart contract related to the transaction. The endorsement node reads the current state of the blockchain and returns the state to the client program after signing the state and the simulated transaction result.

#### 3.4.2. Organized into Blocks

The client program packages the original transaction data and the transaction data signed by the endorsing node and broadcasts it to all organizational nodes. They organize node clusters to continuously receive transaction data from each client, execute the improved consensus algorithm based on BFT-SMART in real time between clusters, and organize transactions that have passed the consensus algorithm into a zone after receiving a certain number of transactions or reaching a certain time.

#### 3.4.3. Confirmation and Submission

When a block is completed, the organization node broadcasts the block to all submitting nodes. The submitting node verifies the timeliness and legality of the block and then uses the transaction data in the block to update the local copy of the blockchain. The specific method is to verify the legitimacy and format of the original transaction information, then read the blockchain state recorded by the endorsement node when the transaction was submitted, and compare it with the current blockchain state. After all transactions are verified, the block is added to the tail of the blockchain saved by the node itself, and the current blockchain state is updated using the data obtained by the previous simulation execution of the endorsement node. Finally, all submitting nodes individually notify the client whether the transaction was successfully submitted. All nodes reach a consensus on the transactions contained in the block and ensure synchronization.

### 3.5. NTRU Algorithm for Multi-Module Blockchain System

NTRU is a public key encryption algorithm based on the difficult problem of lattice theory SVP. Its encryption and decryption only involve addition and subtraction and modular multiplication on polynomials, so it has the characteristics of simplicity, fast calculation speed, and small storage space. In addition, the SVP problem cannot be accelerated by parallel computing in principle and belongs to the category of operations that quantum computing is not good at. This gives the NTRU algorithm a natural anti-quantum computing feature.

The intermediate polynomial of the NTRU decryption process is

(1) $$\begin{array}{c} a\left(x\right)=K_{\text{pri}}\Theta e\left(x\right)\mathrm{mod}q. \end{array}$$

It can be further transformed into

(2) $$\begin{array}{c} a\left(x\right)=K_{\text{pri}}\Theta m\left(-x\right)\left(\mathrm{mod}q\right)-r\left(x\right)\Theta pg\left(-x\right). \end{array}$$

According to the NTRU algorithm decryption rules, we can get

(3) $$\begin{array}{c} a\left(x\right)=r\left(-x\right)\Theta pg\left(x\right)-m\left(x\right)\Theta K_{\text{pri}}. \end{array}$$

When decrypting, let *k*_*i*_ be the coefficients of the polynomial:

(4) $$\begin{array}{c} a\left(x\right)=K_{\text{pri}}\Theta r\left(-x\right)\mathrm{mod}q\Theta pg\left(-x\right). \end{array}$$

Among them, the coefficient of *a* (*x*) is within the range of (−*q*, *q*). At this time, if the value of the *k*_*i*_ parameter is not appropriate, an over-distance error will occur.

The adjustment of public parameters will affect the anti-brute-force attack performance of NTRU and reduce the security of the algorithm. Therefore, adjusting the algorithm parameters according to different practical application scenarios to minimize the probability of decryption failure on the premise of ensuring the security of the NTRU algorithm is a work worthy of research. This section will summarize the existing work on the optimization and adjustment of NTRU algorithm parameters and analyze the feasibility, advantages, and disadvantages of the existing schemes. And according to the application scenario of this paper, a parameter selection of NTRU algorithm suitable for consortium blockchain is proposed.

CS attack is based on the difficult problem of SVP in lattice theory, and it is beyond the scope of this paper to recover the private key through lattice reduction algorithm. The brute-force attack and decryption failure attack can be intervened by presetting the NTRU algorithm parameters. This section will analyze these two attack methods, respectively, and propose a parameter selection that theoretically guarantees the security of the NTRU algorithm and makes it suitable for consortium blockchain scenarios.

The attack against the failure of decryption of the NTRU algorithm is based on unreasonable NTRU parameter settings, and the algorithm output in the case of decryption failure is statistically analyzed to recover part of the secret key information. For this type of attack, the algorithm parameters are preset so that the coefficients of the key polynomial for decryption must be within the value range of (−*q*, *q*).

According to the key generation and encryption operation principle of the NTRU algorithm, to recover the private key polynomial f(x) ∈ *T* (*d*_*1*_: *d*_*2*_), it is necessary to try all possible polynomial combinations using a brute-force attack, and the number of attempts can be recorded as *T*_*N*_:

(5) $$\begin{array}{c} T_{N}=\left(\begin{array}{c} d_{1}\\ N \end{array}\right)\left(\begin{array}{c} N-d_{1}\\ qd_{2} \end{array}\right),\\ \left\{\begin{array}{c} d_{1}=\frac{qN}{3-x},\\ d_{2}=\frac{qN}{3-y},\\ Q_{N}=\frac{3\left(N-d_{1}\right)\left(N-d_{2}\right)}{N!}. \end{array}\right. \end{array}$$

When the NTRU algorithm parameter conditions are set to satisfy *d* = *N*/3 and *q* > 6*N* + 3, the security of the NTRU algorithm can be increased to the highest level without decryption failure. In view of the influence of parameter setting on the performance of NTRU algorithm, this paper makes the following inferences based on the principle of NTRU algorithm:

First of all, the time-consuming operation of the NTRU algorithm is mainly in the modular multiplication operation and the private key modular inverse operation. The time complexity of the modular multiplication operation has nothing to do with the parameter *q*, so setting the parameter *q* will not increase the convolution modular multiplication operation too much.

Secondly, the main idea of the modulo inverse algorithm of NTRU private key is the extended Euclidean algorithm. The time complexity of one iteration of the algorithm is *O* (*n*2), so the worst time complexity of the key generation algorithm is *O* (*n*3), so the parameter *d* has little effect on the algorithm.

Therefore, from a theoretical point of view, properly adjusting the parameters *q* and *d* of the NTRU algorithm will not greatly affect the overall operation speed of the NTRU algorithm, but appropriate parameter selection can improve the anti-attack performance of the algorithm.

## 4. Results and Analysis

### 4.1. Analysis of Comprehensive Cold Chain Logistics Efficiency

**Figure 3:**
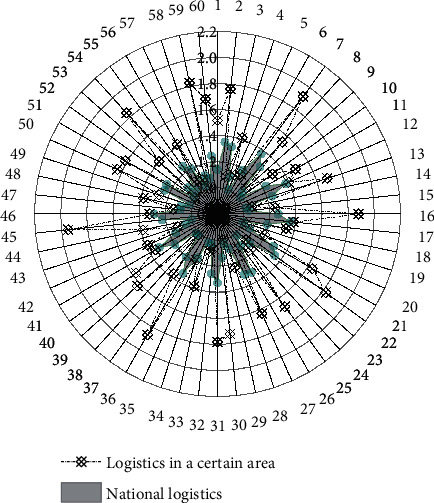
2017–2021 (60 months) agricultural product cold chain logistics compensation output ratio in a certain region.

We calculate and analyze the cold chain logistics efficiency of agricultural products in 60 decision-making units from 2017 to 2021. After sorting out the results, the output ratio of cold chain logistics remuneration for agricultural products in a certain region from 2017 to 2021 is obtained, as shown in Figure 3.

When the comprehensive cold chain logistics efficiency value is equal to 1, it means that the allocation of resources is relatively reasonable, the input and output are in the best condition, and the comprehensive cold chain logistics efficiency is effective at this time. When the cold chain logistics efficiency value is greater than 0.9 and less than 1, it means that the resource allocation is slightly unreasonable, and the input and output have not reached the optimal state. At this time, the comprehensive cold chain logistics efficiency is weak and effective. When the cold chain logistics efficiency value is less than 0.9, it means that the allocation of resources is obviously unreasonable, and there is a large gap between the input and output and the optimal situation. At this time, the comprehensive cold chain logistics efficiency is invalid.

From a vertical comparison, the comprehensive cold chain logistics efficiency of agricultural products in a certain region in 2018 was 1, indicating that the cold chain logistics efficiency of agricultural products in 2018 was at the forefront of production, resource allocation was optimal, and both input and output were in the best condition. In 2019, the comprehensive cold chain logistics efficiency values were all less than 0.9, indicating that the comprehensive cold chain logistics efficiency was ineffective, indicating that the allocation of cold chain logistics resources in the past four years was not reasonable enough, resulting in a significant gap between input and output and the best situation.

**Figure 4:**
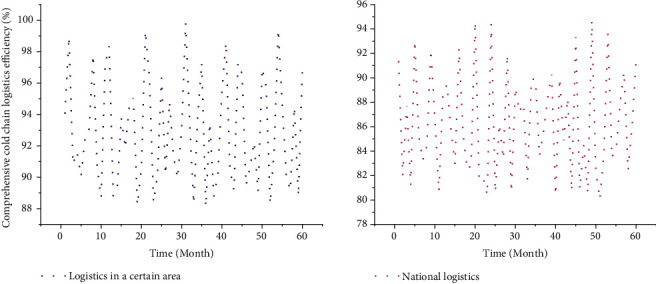
Comparison of the comprehensive cold chain logistics efficiency of agricultural products in a certain region and the whole country from 2017 to 2021 (60 months).

From a horizontal comparison, according to the comprehensive cold chain logistics efficiency values of 60 decision-making units from 2017 to 2021, the average value of the comprehensive cold chain logistics efficiency of each decision-making unit was obtained. The comprehensive cold chain logistics efficiency of agricultural product cold chain logistics in a certain region is 0.93, which is greater than the average annual comprehensive cold chain logistics efficiency of 0.87 in the country, indicating that the current agricultural product cold chain logistics in a certain region is at a relatively high level of development. The data are compared month by month to obtain Figure 4.

It can be seen from Figure 4 that in the five years from 2017 to 2021, the comprehensive cold chain logistics efficiency of agricultural products in a certain region is higher than the national average comprehensive cold chain logistics efficiency of agricultural products. In the 30th month, the comprehensive cold chain logistics efficiency of agricultural products in a certain region is 1, which is in the optimal state. It is very important to adjust the allocation of agricultural products cold chain logistics resources in a certain region in time and improve the comprehensive cold chain logistics efficiency of agricultural products in a certain region.

### 4.2. Efficiency Analysis of Pure Technology Cold Chain Logistics

If the pure technical cold chain logistics efficiency is equal to 1, it means that the use of input resources is efficient at the current technology level. If the pure technical cold chain logistics efficiency is less than 1, it means that the use of input resources is not effective under the current technical level, which means that the technical level cannot meet the needs of industrial development at this time.

In terms of vertical comparison, the efficiency value of pure technology cold chain logistics in 2018 was 1, indicating that the cold chain logistics of agricultural products in a certain region was efficient in pure technology cold chain logistics, the level of input resource utilization was relatively high, and the overall technology level could support a certain region. In the remaining years, the efficiency values of pure technology cold chain logistics were all less than 1, indicating that these years did not reach the efficiency of pure technology cold chain logistics, and the technical level needs to be adjusted.

**Figure 5:**
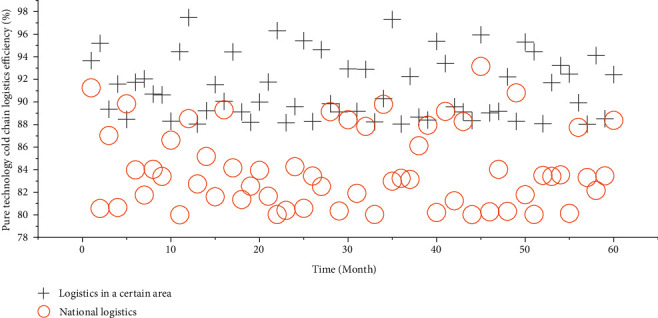
Comparison of the efficiency of pure technology cold chain logistics of agricultural products in a region and the whole country from 2017 to 2021 (60 months).

From a horizontal comparison, according to the pure technology cold chain logistics efficiency values of 60 decision-making units from 2017 to 2021, the average value of pure technology cold chain logistics efficiency of cold chain logistics of each decision-making unit was obtained. The average efficiency of pure technology cold chain logistics for agricultural products in a certain region is 0.93, which is higher than the national average of 0.86 for pure technology cold chain logistics.  Figure 5 is obtained by comparing the efficiency of pure technology cold chain logistics of agricultural products in a certain region with the national value in the past five years.

It can be seen from Figure 5 that in the five years from 2017 to 2021, the pure technology cold chain logistics efficiency of agricultural product cold chain logistics in a certain region is higher than the national average pure technology cold chain logistics efficiency. However, in a certain region, the efficiency value of pure technology cold chain logistics of agricultural products has not reached 1. On the whole, the national agricultural product cold chain logistics needs to be further optimized in terms of technical input and management level, such as introducing advanced cold chain technology in technical input, strengthening the construction of cold chain logistics informatization, and updating related infrastructure.

### 4.3. Analysis of Scale Cold Chain Logistics Efficiency

If the scale cold chain logistics efficiency is equal to 1, it means that the production scale reaches the optimal state under the current conditions; if the scale cold chain logistics efficiency is less than 1, it means that the production scale is not suitable for the cold chain logistics efficiency, and the scale is invalid at this time.

From a vertical comparison, the scale of cold chain logistics efficiency in 2018 was 1, the scale of agricultural cold chain logistics in a certain region was effective in that year, and the return to scale was unchanged. This shows that in 2018, a certain region has achieved a good plan in terms of the scale structure of agricultural product cold chain logistics, which meets the needs of development and maximizes scale benefits. In the rest of the years, the scale cold chain logistics efficiency value is less than 1, but all are greater than 0.9, indicating that the scale of agricultural cold chain logistics in a certain region in these years is in a weak and effective state, and the industrial scale is slightly unreasonable.

**Figure 6:**
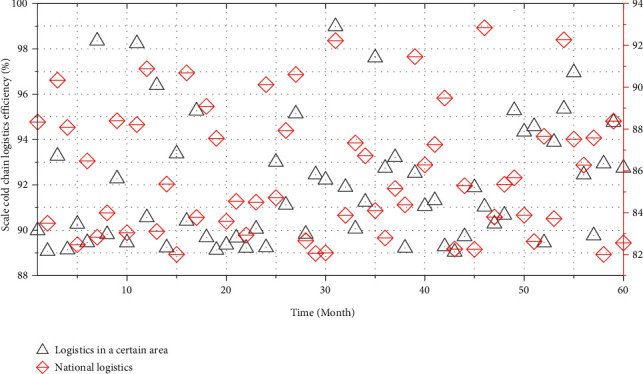
Comparison of cold chain logistics efficiency of agricultural products in a certain region and the whole country from 2017 to 2021 (60 months).

From a horizontal comparison, according to the scale cold chain logistics efficiency values of 60 decision-making units from 2017 to 2021, the average cold chain logistics efficiency of cold chain logistics of each decision-making unit was obtained. The scale cold chain logistics efficiency of agricultural product cold chain logistics in a certain region is 0.95, which is higher than the average annual scale cold chain logistics efficiency of 0.93 in the country, indicating that the scale cold chain logistics efficiency of agricultural product cold chain logistics in a certain region is at a high level.  Figure 6 is obtained by comparing the cold chain logistics efficiency of agricultural products in each year in a certain region with the average value of each year in the country.

It can be seen from Figure 6 that in the five years from 2017 to 2021, the scale of cold chain logistics efficiency of agricultural products in a certain region is generally higher than the efficiency value of national scale cold chain logistics. However, at present, a certain region should continue to increase the investment in the scale of the cold chain logistics industry to match the existing technology, so as to improve the utilization of resources and maximize the scale benefit.

### 4.4. Input Redundancy Analysis

**Figure 7:**
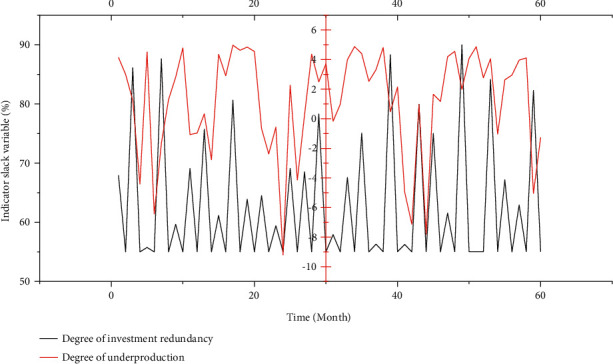
Slack variables of input and output indicators of agricultural cold chain logistics in a certain region.

According to the DEA analysis results, it is possible to find out the redundant input and insufficient output during the period when the cold chain logistics efficiency of agricultural products in a certain region is ineffective, so as to improve it.  Figure 7 shows the results of the input and output indicators of a region from 2017 to 2021.

From Figure 7, it can be seen that the distribution of agricultural product cold chain logistics resources in a certain region is relatively reasonable, there are no redundant input and insufficient output, and the agricultural product cold chain logistics resources in a certain region have been reasonably utilized.

In 2017, the investment in logistics-related fixed assets and logistics-related employees had redundant investment, indicating that there were relatively too many logistics employees in a certain region and excessive cold chain logistics-related construction and investment. As a result, cold chain logistics resources are not fully utilized, resulting in low efficiency of cold chain logistics. In 2017, the return to scale of the cold chain logistics of agricultural products in a certain region is increasing. According to the increasing return to scale, the output will increase at a higher proportion after increasing the input. By adjusting the structure and proportion of resource input, the full use of resources can be achieved.

From the perspective of input indicators, there are varying degrees of redundancy in logistics-related fixed asset investment, logistics-related employees, and per capita cold storage capacity. From the perspective of output indicators, there is insufficient output in the growth elasticity of cold chain logistics. It shows that the use of input resources in a certain region in the past three years has not been reasonable enough, and the input resources have not been fully effective, which not only leads to the waste of input resources, but also makes the output insufficient, which affects the efficiency of the cold chain logistics of agricultural products in a certain region. Therefore, a certain region should improve the ability to allocate resources and management level, and optimize the development of the existing cold chain logistics.

### 4.5. Dynamic Analysis of Cold Chain Logistics Efficiency

**Figure 8:**
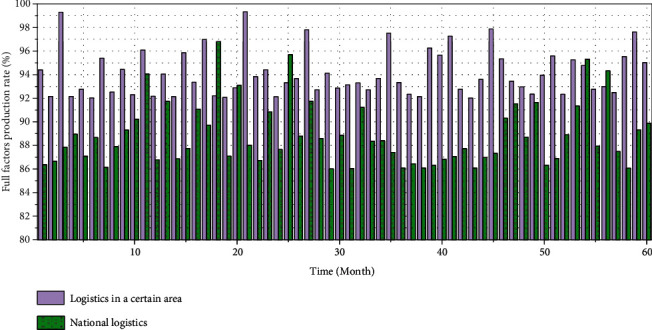
2017–2021 (60 months) changes in total factor productivity of agricultural product industry cold chain logistics in a certain region.

Based on the static analysis of DEA's cold chain logistics efficiency, this paper adopts the Malmquist index analysis method to dynamically analyze the cold chain logistics efficiency of agricultural products in a certain region from 2017 to 2021. We use DEAP2.1 software to calculate total factor productivity changes, technical cold chain logistics efficiency changes, pure technical cold chain logistics efficiency changes, scale cold chain logistics efficiency changes, and technological changes, and decompose the reasons for such changes. The results of Malmquist index analysis are sorted to obtain Figure 8.

As can be seen from Figure 8, in the period of 2017–2018, the technical cold chain logistics efficiency, technological change, pure technical cold chain logistics efficiency, scale cold chain logistics efficiency, and total factor productivity of agricultural product cold chain logistics in a certain region are all greater than 0.9, which indicates that in this stage, the cold chain logistics industry of agricultural products in a certain region is on the rise in technology and scale, the level of total factor productivity is improving, and the allocation of resources is relatively reasonable.

According to the change of total factor productivity, the reasons for the change of total factor productivity are analyzed. From 2018 to 2019, the technical cold chain logistics efficiency change was less than 1, and the technical change was greater than 0.9, indicating that the technical cold chain logistics efficiency was decreasing during this time period, and the main reason for the decline in total factor productivity was the technical cold chain. The logistics efficiency is low; during the period of 2019–2021, the change of technical small cold chain logistics efficiency is greater than 0.85, and the technical change is less than 1, indicating that the efficiency of technical cold chain logistics is constantly improving in this stage. The actual output is constantly increasing, and the lack of technological input leads to technological regression, which is the main reason for the decline of total factor productivity. On the whole, the mean value of the change of technical cold chain logistics efficiency is less than 1, which indicates that the technical cold chain logistics efficiency of agricultural product cold chain logistics industry in a certain region has declined during this research stage.

According to the change of technical cold chain logistics efficiency = pure technical cold chain logistics efficiency change × scale cold chain logistics efficiency change, the change trend of technical cold chain logistics efficiency can be analyzed. The change of technical cold chain logistics efficiency is basically the same as that of pure technical cold chain logistics efficiency. Therefore, it is believed that the main reason for the change of technical cold chain logistics efficiency is the change of pure technical cold chain logistics efficiency.

## 5. Conclusion

This paper proposes a multi-layer blockchain model that supports hierarchical authorized transactions of massive data. The alliance chain network is deployed between the main body and the main body, and the private chain network is deployed inside the main body. By introducing the authority management and identity authentication mechanism, the identity certificate and transaction certificate are issued for each node between the alliance chains, and the access control function in the transaction process is realized by means of the channel pipeline mechanism. The blockchain platform adopts the method of off-chain storage to store real data in the IPFS cluster server built by the alliance. The blockchain stores the index hash of the file, which greatly saves the storage space consumption of the blockchain. In 60 months, the comprehensive cold chain logistics efficiency reached an effective state, the allocation of input resources was reasonable, and the resources were fully utilized. Through the analysis of input redundancy, it can be seen that there is a certain redundancy in logistics-related fixed asset investment, logistics-related employees, and per capita cold storage capacity. However, there is no shortage of output from 2019 to 2021, indicating that a certain region has a better technical level and management level in developing agricultural cold chain logistics, the utilization of resources is relatively sufficient, and the efficiency of cold chain logistics is high. From 2018 to 2021, the total factor productivity of the cold chain logistics industry of agricultural products in a certain region is relatively high, mainly due to the better efficiency of technical cold chain logistics. By decomposing the change of technical cold chain logistics efficiency, it is found that the change of technical cold chain logistics efficiency is caused by the change of technology application level, so the technology application level is the main reason for the change of total factor productivity. It shows that the technical application level related to the cold chain logistics of agricultural products in a certain region is obviously relatively sufficient from 2018 to 2021.

## Figures and Tables

**Table 1:** Data structure design of private chain smart contract.

User info	Department name	Account public key	Account private key	Account address
Data information	File hash generated based on IPFS	File account	Data ID	Account data
Operational information	Serial number	Account address	Generation time	Operation type

**Table 2:** Data structure design of alliance chain smart contract.

Trading information	Operational information
Trading status	Transaction new release; transaction validation
Transaction data ID	Generation time
Transaction hour	Operation type
Initiating transaction address	Serial number
Accepting counterparty address	Account address

## Data Availability

The data used to support the findings of this study are included within the article.
